# Perspectives on “Novel” Techniques for Designing Age-Friendly Homes and Neighborhoods with Older Adults

**DOI:** 10.3390/ijerph17051800

**Published:** 2020-03-10

**Authors:** Katherine Brookfield, Iain Scott, Anthea Tinker, Catharine Ward Thompson

**Affiliations:** 1 Environment and Geography Department, University of York, York YO10 5NG, UK; 2 Edinburgh School of Architecture and Landscape Architecture, University of Edinburgh; Edinburgh EH1 1JZ, UK; iain.scott@ed.ac.uk; 3 Institute of Gerontology, Department of Global Health & Social Medicine, King’s College London, London WC2B 4BG, UK; anthea.tinker@kcl.ac.uk; 4 OPENspace Research Centre, University of Edinburgh, Edinburgh EH1 1JZ, UK; c.ward-thompson@ed.ac.uk

**Keywords:** aging and housing, domestic design and technology, co-design, participatory design

## Abstract

Worldwide, growth in the older population creates a pressing need to develop supportive environments that enhance quality of life as people age. Too often, built environments present barriers and challenges to older adults that compromise independent living and adversely affect health and life outcomes. Designing homes, buildings, and neighborhoods with older adults, through exercises in participatory or co-design, could help ensure that environments are better able to facilitate healthy aging. However, while it is potentially advantageous to involve this age group in environmental design decisions, doing so can be difficult. Analysis of and guidance on effective ways to involve older adults in these activities could make the challenge easier. With this aim in mind, this article provides critical perspectives on eight “less traditional” engagement techniques—walking interviews, photovoice, photo-elicitation, Talking Mats^®^, participatory mapping, drawing, model-making, and the “Design Fair”. Insights into the strengths and limitations of these techniques, gained from observation of their use in participatory design activities, as well as feedback collected from older co-design participants, are presented. The article concludes by offering a number of practical recommendations for those interested in designing age-friendly homes and neighborhoods with older people.

## 1. Introduction

Worldwide, growth in the older population [1] creates a pressing need for “age-friendly” environments that optimize opportunities for social and economic participation, health, and security in order to enhance quality of life as people age [2]. The goal of creating age-friendly environments is a strategic objective in the World Health Organization’s [3] Global Strategy and Action Plan on Aging and Health (2016–2030). It also relates to many of the United Nations (UN)’s [4] Sustainable Development Goals and to various national and international strategies and initiatives, such as the European Union (EU)’s new “Smart Healthy Age-Friendly Environments” (2019–2023) policy development program, and the recently launched “Center for Active Aging and Innovation”, established by the Association of Southeast Asian Nations [5]. The significance attached to creating age-friendly environments points to the scale of the problems we face with regard to our current environments. All too often, built environments present barriers and challenges to older adults that adversely affect their quality of life, opportunities for independent living, health, and life outcomes [2,3]. Involving older adults in decisions about the design of homes, buildings, and neighborhoods could help ensure that environments are better able to support healthy aging. Indeed, subscribing to this logic, the World Health Organization [2] involved older adults in the preparation of its influential “how-to” guide on developing age-friendly environments: “Global age-friendly cities and communities: a guide”.

The value of involving “users” in the design of environments, systems, services, and products is acknowledged by diverse disciplines and areas of work [6], from health [7] to urban planning [8], social care [9], and information technology [10]. Including users in the design process as designers [6], an activity variously termed “participatory design” or “co-design”, is thought to help ensure outputs better reflect users’ requirements and ambitions [11], which in turn might increase an output’s accessibility [12] and encourage its adoption/use. For example, recent research found that older adults often delay introducing home adaptations such as handrails and ramps, which can support independent living, because they find the design of these items stigmatizing and aesthetically unappealing [13,14,15]. Moreover, it is argued that it is intrinsically right to include those who are affected by a decision in the process of making that decision [16], a sentiment captured in the refrain, commonly used by disability rights advocates, “nothing about us without us” [17].

Although it might be desirable and advantageous to involve older adults in decisions about the design of housing, buildings, and neighborhoods, doing so can be challenging. The need for non-hierarchical, inclusive situations, where power is ceded from designers to users, and the designer is no longer the primary source of creativity [16], runs contrary to traditional ways of working within design disciplines [18,19,20]. In environmental planning, a focus on the wider “public interest” can result in minority interests, such as those of older adults, being marginalized [20]. Older adults may require tailored engagement techniques [19,21] that respond to age-related health conditions [22], while there is some evidence to suggest that older adults can struggle to envision less tangible issues when reflecting on design problems [23]. Despite such factors, some older adults do successfully participate in environmental design decisions. For example, following an extensive review of the social and civic participation literatures, Brodie and colleagues [24] concluded that older adults are more likely than younger adults to participate in local decisions, like decisions on planning and land-use regulation. However, older adults are a diverse population and, across this age group, participation in environmental decision-making is uneven. For instance, it was specifically White, better educated, richer, middle-class older males who were identified, by Brodie and colleagues [24], as being more likely to participate in local decision-making. Engagement techniques that help encourage participation from all quarters of the older population would seem, therefore, important. In this context, “novel” techniques that offer alternative ways to engage might be particularly valuable. Non-verbal techniques, such as drawing or model-making, might, for instance, be especially suited to facilitating the participation of older adults who, for whatever reason, are less confident speaking or writing [25].

To further aid older adults’ involvement in environmental design decisions, we provide critical perspectives on eight, relatively novel engagement techniques: walking interviews, photovoice, photo-elicitation, Talking Mats^®^, participatory mapping, drawing, model-making, and the “Design Fair”. We consider their merits as potential tools for involving older adults in these types of decisions. While there are evaluations of the pros and cons of many of these techniques, particularly so in the case of Talking Mats^®^ (see References [26,27,28]), these assessments rarely reflected on if and how a technique could be used as an effective mechanism for incorporating older adults’ voices into decisions on the design of our environments. Several studies considered the potential for novel techniques, such as drawing and model-making, to be used as tools for involving older adults in decisions about the design of information technology products [12,29,30]. However, there are very few examples of studies that evaluated novel techniques for environmental co-design with older people. Fang and colleagues [20] considered how group walks and participatory mapping could help older adults and service providers collaborate in the co-creation of place-based interventions in a large, multicultural Canadian city. However, with a focus on service provision, there was little engagement of older adults in the co-design of the physical environment itself. Responding to this gap in the literature, we draw on observations of the eight techniques as they were employed at various participatory design events, as well as on feedback collected from the older adults (typically over 60 years old) who participated in these events, to reflect on the value each might have as a mechanism for including older people in environmental design decisions.

The rest of the article is organized into four sections. The next section describes the eight techniques, the contexts in which they were employed and observed, and the methods used to collect feedback from the older adults who participated in the co-design events. The results and discussion sections present perspectives on the value the techniques might have as ways for involving older adults in environmental design decisions. The conclusions offer a small number of practical recommendations for those interested in designing age-friendly homes and neighborhoods with older people.

## 2. Materials and Methods

We organized a series of participatory design events across the United Kingdom (UK) over a three-year period. These included three co-design workshops, three design review events, and 22 one-to-one interactions with diverse older adults. Within these, we implemented a collection of more “traditional” techniques, including structured interviews and focus groups, as well as more “novel” engagement techniques. For the reasons previously discussed, our focus here is exclusively on the novel techniques.

The co-design workshops and design reviews were held in central Manchester in Northwest England, Hackney Wick in East London, and Kirkwall on the Orkney Islands, an archipelago off the north coast of mainland Scotland. All workshops and reviews were place-specific. The Manchester co-design workshop and design review focused on developing environmental designs for a more age-friendly district of central Manchester, the Hackney Wick events focused on designing a more age-friendly Hackney Wick, and the Kirkwall events focused on designing a more age-friendly Kirkwall and surrounding island environment. The one-to-one interactions were held in Edinburgh. These focused on a participant’s own home and various hypothetical indoor and outdoor environments, investigating how these places could be more or less “age-friendly”. With the participant’s permission, these one-to-one interactions were audio-recorded and subsequently transcribed.

The four locations—central Manchester, Hackney Wick, Edinburgh, and Kirkwall on the Orkney Islands—were chosen to provide a wide variety of geographic, environmental, and socioeconomic contexts. The locations ranged from a dense urban neighborhood in the UK’s largest city (Hackney Wick) to a small market town on a remote, largely rural island (Kirkwall). Table 1 provides headline demographic data for the four locations.

As shown in Table 2, the number of participants, the participant characteristics we recruited for, and event length differed between events and locations. Varying these factors provided opportunities to observe the value, effectiveness, limitations, and so forth of events of differing lengths, sizes, and participant compositions. Table 2 also shows how the collection of engagement techniques employed varied between events and locations, as well as how our events were staggered over a three-year period. Varying the techniques between events enabled consideration of a larger number of techniques. An event’s environmental context, findings from the participatory design and research methods literature, and an interest in piloting new methods and testing “proven” techniques in different settings informed the choice of techniques employed at each event. For example, techniques that appeared “effective” in some way were carried over from one event to the next, circumstances allowing (e.g., venue size), to explore if their “effectiveness” was replicated with different sets of participants. Time pressures and concerns about minimizing the demands placed upon older participants meant that several engagement techniques were often combined within single “integrated activities”. For example, at the co-design workshops held in central Manchester and Hackney Wick, we combined the techniques of walking interviews and photovoice in a single “Walk and Talk” activity. This meant that, at each location, participants took one walk around the neighborhood in which we worked and, on this walk, completed a walking interview and, addressing the concerns of photovoice, took photographs of the environments traversed and then discussed these photographs at the end of the walk. Participants were not, therefore, asked to undertake two walks—one walk for a walking interview and a second walk for photovoice.

Individuals provided informed consent; our work was approved by a Research Ethics Committee at the second author’s institution and, for the one-to-one interactions in Edinburgh, by the National Health Service (NHS) West of Scotland Research Ethics Committee (REC reference: 13/WS/0183). As appropriate, our work was conducted in accordance with the Declaration of Helsinki.

The first and second authors and groups of (usually postgraduate) architecture and landscape architecture students from the second author’s institution, identified here as “design students”, facilitated the co-design workshops and design reviews, noting evidence of the value of including facilitators and designers in co-design activities [12,21,30,31]. The first author led the one-to-one interactions. The eight engagement techniques identified in Table 2 and employed in the co-design workshops, design reviews, and one-to-one interactions are described below.

*Photovoice* involves individuals using photography to record some aspect of their life, like the assets and needs of their neighborhood [32]. The photographs and their meanings are then discussed and critically reflected upon in one-to-one conversations and/or in groups. The method can facilitate the participation of often-excluded groups, such as people who cannot read or write [32]. We used the technique at our co-design workshops in central Manchester and Hackney Wick. Older adults, working with design students, walked around local neighborhoods in these locations and photographed “items of interest”, such as perceived positive and negative neighborhood attributes. Following the walk, they discussed these photographs with the design students.

*Model-making* is a creative, visual method of design communication [33]. We employed this method at our co-design workshops in Hackney Wick and Kirkwall. For the Hackney Wick workshop, design students made, in advance, a simple three-dimensional base structure from modeling foam that represented the neighborhood we worked in. Within the workshop, older adults worked alongside the design students to revise the model, removing elements (e.g., buildings) they disliked and using modeling foam, plasticine, and card to introduce new structures and environmental interventions. For the Kirkwall workshop, older adults worked with design students to create a prototype “ideal” home for older adults living on Orkney that responded to the landscape and older adults’ (varied) requirements. Both workshops produced rough, conceptual models [33].

*Walking interviews* combine participant observation with interviewing and involve interaction “on the move” [34]. Insights into the body in motion within a given environmental setting are possible, while the format of a walking interview, i.e., the interviewer and interviewee walking along side-by-side, can disrupt the power relations often found within “traditional” interviews. We used the method at our co-design workshops in central Manchester and Hackney Wick. At each location, older adults walked around a local neighborhood with design students and discussed their impressions of the environments encountered.

*Participatory mapping* entails the production of a spatial map in collaboration with members of a community, often through reference to local knowledge and resources [35]. Maps may be constructed using multiple materials, from simple paper and pens through to online mapping or GIS (geographic information system). The process of creating and negotiating the map content is as important as the finished map. We used the method at our workshops in Hackney Wick and Kirkwall. Older adults annotated large Ordinance Survey maps related to the areas we worked in, noting items/areas that elicited positive and negative emotional responses, which formed aids and challenges to healthy aging and so forth.

*Drawing* activities can be diverse, ranging from abstract or representational sketches to precise diagrams. Drawing can allow ideas to be investigated quickly [36] and details to be elaborated [12]. We used the method at our Hackney Wick workshop. Older adults, working with design students, sketched out their impressions of the neighborhood within which we worked, identifying items they liked and disliked, which provoked a positive or negative emotional response, and so on.

“*Design Fairs*” were held in Hackney Wick and Kirkwall. At these events, the design students who participated in the co-design workshops held in these locations staffed “stalls” that displayed models and drawings of age-friendly home and neighborhood designs, which they had created. These reflected the issues and rough designs previously identified and co-created with older adults at the co-design workshops. Invited older adults, including many who attended the co-design workshops, moved between the stalls talking to the students about their proposals and suggesting how they could be further refined to be yet more “age-friendly”. Such critiquing of design is one of the most common mechanisms via which “non-designers” are included in the design process [12].

*Photo-elicitation* involves introducing images into an interview to inspire reflection and comment [37]. Images can be provided by the interviewer or the interviewee. We used this method in our one-to-one interactions with older adults in Edinburgh. The older adults were shown six researcher-generated photographs of different outdoor environments. Half showed environments containing features and items that, according to research by the World Health Organization [2], are viewed favorably by older people (e.g., people interacting with one another), and half contained features and items that are viewed negatively (e.g., litter). Questions explored participants’ perceptions of these different features and items. We also used this method in our Manchester co-design workshop. The design students showed the older participants images of the neighborhood in which we worked and asked questions about their perceptions of and attitudes toward the places and environmental details represented.

*Talking Mats^®^* is a low-technology, picture-based communication framework developed to help individuals with communication difficulties express their views [26]. The method requires individuals to consider a picture that illustrates an activity, item, relationship, and so forth and to indicate their views toward it by placing it somewhere along a visual scale that captures some concept of interest, such as preference [26]. We used the method in our one-to-one interactions with older adults in Edinburgh. We developed a visual scale that engaged with the concept of importance, running from not important to important. The pictures we used illustrated 17 features of the home and of outdoor environments that were identified by the World Health Organization [2] as necessary components of an age-friendly home and city. Features were varied ranging from storage space to kitchen facilities, and from green space to smooth pavements.

Wishing to understand the potential value of the eight techniques as mechanisms for involving older adults in environmental design decisions, we asked participants at the central Manchester events and at the Hackney Wick co-design workshop to complete a short survey; the survey employed at each event was tailored to the content of that event. The surveys explored thoughts on how enjoyable the techniques were, how effective they were for exploring participants’ views on the design of environments, whether opportunities were provided for participants to discuss things that they felt were important to the design of environments, and how easy the techniques were to understand and to engage with. Usually, questions took the form of statements with participants indicating their degree of agreement with the statement using a five-point Likert scale. It was anticipated that it would be easier for participants to recall the different “integrated activities” they completed rather than the individual techniques incorporated within these activities. Consequently, the surveys explored reflections on the “integrated activities” and, thus, did not produce data on participants’ appraisals of individual techniques. Table 3 and Table 4 present the results of the surveys conducted at the central Manchester and Hackney Wick co-design workshops. Results from the Manchester design review survey are not presented as this event did not incorporate any of the eight engagement techniques that are the focus of this article. As shown in Table 2, feedback was also collected from participants informally at the various events and one-to-one interactions. Participants were invited to share their views in informal conversations with the first and second authors and to use anonymous “comment cards” (post-it notes) to jot down thoughts on the events and techniques.

In addition to participant feedback, observation was used to examine and evaluate the different techniques. Observation is an established and valuable method for examining and evaluating practices, services, and behaviors [38,39,40]. For example, in education, peer observation and critical self-reflection are employed to evaluate teaching technique and student engagement [41,42], while, in health research, observation is used to assess diverse items such as nature-based therapeutic interventions [43], dementia care and dementia care services in different settings [39,44,45], and the allocation of pre-hospital medical assistance and resources [46]. To help determine the potential value of the eight techniques as mechanisms for involving older adults in environmental design decisions, the first author observed and made field notes during and following the participatory design events on factors such as levels of engagement, group dynamics, successes, and areas for improvement. Field notes were also made immediately after each one-to-one interaction capturing observations on researcher–participant rapport, perceived levels of interest/engagement in the techniques, any distractions/interruptions that occurred during the interaction, and so forth.

Drawing on participant feedback and observations of the techniques in use, the next section reflects on the possible value of the eight techniques as mechanisms for including older adults in environmental design decisions.

## 3. Results

Participant feedback, captured through the self-complete surveys (Table 3 and Table 4), informal conversations, and anonymous comment cards, as well as observation of the techniques in use, suggests that all eight techniques might offer potentially valuable ways of involving older adults in environmental design decisions.

Walking interviews appeared to be a particularly valuable technique. Walking around a neighborhood while informally discussing its attributes was an enjoyable activity for the majority of older adults who participated in our events, and the activity produced rich, first-person accounts of environmental aids and challenges that could inform design decisions. Immersion within the environment during the walk meant that a huge array of environmental details, which might not have been mentioned in a traditional interview, were encountered and subsequently discussed. Older adults believed that the method allowed them to discuss topics that they felt were important to the design of environments, and they generally believed that it was an effective method for exploring their views. Asking older adults to take photographs of the environment during the walk, and following this up with a focused discussion about the photos taken, which constituted our use of photovoice, also proved popular with the participants and produced extensive material that could inform design decisions. More negatively, individuals with mobility limitations were identified by some older participants as being potentially less able to participate in these methods. However, as shown in Table 4, participants themselves found the methods “easy” to engage with indicating that they personally did not experience any difficulties around participation. Developments in online mapping services, which provide 360-degree imagery of street-level scenes, such as Microsoft’s StreetSide^®^ (Microsoft, Redmond, WA, USA) or Google Street View™ (Google Inc., Mountain View, CA, USA), could reduce possible barriers to participation that might be experienced by individuals with limited mobility. It might be possible to use this technology to engage participants in a “virtual” walking interview using the technology to follow a route through a neighborhood while discussing the street-level imagery presented on screen. Furthermore, connecting to the interests of photovoice, screenshots of street scenes selected by the participant could be taken and subsequently discussed.

The two interviewing techniques that incorporated images as part of their method, photo-elicitation and Talking Mats^®^, proved effective at prompting more complex and expansive responses than might have been possible in a traditional interview and appeared popular with the older participants. During the photo-elicitation activity in the one-to-one interactions in Edinburgh, older adults often built on their initial responses to questions, engaging in a form of dialogue with the presented images. Frequently referring back to them, they questioned, challenged, and discussed the content of the images, reflecting on what was shown and speculating on what was not shown, what could have been shown, and so on. For example, in an image showing adults conversing with one another on a street and children playing in the street, participants queried how common these behaviors were, compared contemporary streets to the more “sociable” spaces they remembered from their past, raised safety concerns, their own and those perceived within wider society, about children playing in the street, and discussed how street design can affect behavior. Introducing seating areas set back from the street pavement was identified, for example, as a design intervention that could encourage interaction, as individuals who share a bench might share a conversation. The opportunity to study the images appeared to provide individuals with a “natural” opportunity to pause, reflect, and then present an answer; in traditional interviews, fear of the “awkward silence” can lead to hurried, less considered responses. Older adults found the Talking Mats^®^ technique novel and interesting, while the process of considering each of the 17 elements in turn, placing them on the visual scale, and then discussing their placement, produced an exhaustive account of their views on multiple aspects of the home and of outdoor environments, as well as of relationships between these aspects—the importance of green space relative to pavements, spacious rooms relative to a comfortable ambient temperature, and so on. The pictorial representation of views produced through Talking Mats^®^ appeared to be an aid to both the researcher and the older participant. It ensured that none of the 17 aspects were missed in discussion and, helping to reduce power imbalances, it enabled both parties to pick out aspects to explore further, with discussion becoming less directed by the researcher. Both image-based techniques produced extensive information, including fine-grained detail about the preferred design of different elements of the home and of outdoor environments. In a similar vein, participatory mapping, another image-based technique, provided detailed site-specific information about favored design interventions, their form and scale, environmental details that should be removed/improved within an area, and so on. For example, at the Hackney Wick workshop, participants pinpointed on their maps the locations of graffiti and street art murals that they valued and favored retaining.

Drawing and, to a lesser extent, model-making generated variable levels of engagement from our older participants and seemed to prompt somewhat mixed responses. For example, whereas all participants at the Hackney Wick co-design workshop (who completed the survey) agreed or strongly agreed that the integrated walking interview and photovoice activity (“Walk and Talk”) was enjoyable and easy to participate in, and provided opportunities to discuss topics that were important to the design of environments, they were less unanimous about the merits of the integrated activities that encompassed drawing and model-making. Perhaps anticipating the design students to have superior creative skills, one participant commented that “the students were gifted at drawing and modeling”, there seemed to be a tendency amongst some to defer to the students when it came to presenting ideas in the form of a drawing. Although most participants reported finding it “easy” to engage with the technique (Table 4), there was also some reluctance to engage directly in making a model. At the Hackney Wick co-design workshop, a couple of older adults offered suggestions about how to build the model, but refrained from directly working with the modeling materials. We might speculate that the technique was considered “easy” to engage with because it supported different levels of engagement—from directly making a model to providing guidance to others on model construction.

Models proved adept communication tools when older adults and design students sought to present their visions for age-friendly homes and neighborhoods to others. Employed within the co-design workshops and at the “Design Fair” events in Hackney Wick and Kirkwall, models quickly and simply communicated design ideas, as well as captured people’s attention. At the Manchester design review event, which adopted a “town hall meeting” format, slides and oral presentations rather than models were used to present design ideas. Sometimes, these techniques failed to clearly and engagingly communicate ideas, while the older adults who attended commented that they would have appreciated seeing the proposals presented in model form. The design review events revealed the importance of developing user-centered forms of communication in user-centered design exercises. In design, ideas are often communicated through complex drawings and language that can only be de-codified and understood by individuals with specialist design training [47]. It can be easy to overlook how exclusionary jargon, technical terms, and even a particular style or size of font [48] can be to “non-designers”. Everything from the complexity of visual imagery to the materials used to convey information had to be carefully (re)considered and (re)designed to ensure clear communication at these events and, indeed, in all our interactions with older adults [47].

The Design Fair events provided individuals with the opportunity to manage their own period of participation with a “drop-in” approach adopted to attendance. This approach appeared to encourage wider involvement with attendance at the Hackney Wick (*n* = 18) and Kirkwall (*n* = 11) events being notably higher than at the Manchester event (*n* = 6), where a drop-in approach was not employed. Observing events of differing durations, and observing older adults during events, highlighted clearly how older participants can become tired and disengaged when events span more than a couple of hours. Moreover, it proved difficult to recruit older adults to multi-day workshops with individuals reporting that other commitments—from caring for grandchildren to club or class attendance—prevented their involvement in activities that spanned several days.

## 4. Discussion

Our experiences of implementing the eight engagement techniques largely reflect those reported by others. For example, model-making enabled individuals to present their ideas quickly and directly [49]. Complex or ambiguous ideas were made simple and straightforward while the construction of a model fostered collaboration [50] between older adults and design students. Photovoice uncovered rich, first-person accounts of the neighborhoods in which we worked [51], while walking interviews elicited detailed and potentially more meaningful perceptions of these neighborhoods [52]. Our work suggests that these techniques can be successfully employed with older adults, and that doing so brings similar advantages to those obtained when they are employed with other population groups. However, it also revealed that it might be appropriate to introduce certain modifications when implementing these techniques with an older demographic. Mobility limitations, visual impairments, and various other health conditions can be more prevalent amongst older adults. Mechanisms for involving older people in environmental design decisions must respond to this context. Shorter engagement activities, opportunities for individuals to manage the length of their participation, rest breaks, refreshments, opportunities to engage in different ways (talking, drawing, model-making, etc.) and at different “levels”, and an absence of jargon can and did support older adults’ involvement. Similar accommodations have been identified by others as preferred mechanisms for facilitating successful co-design with older users [21,22,23,29,53].

Matching the experience of some researchers, our techniques, particularly the model-making activities, produced a range of creative and novel design proposals. The three-dimensional, tactile qualities of the models [33], combined with the “rough and ready” approach adopted when creating them, seemed to unlock creativity in some older participants. Often, the older adults surprised the design students with the boldness of their design suggestions [47]. At the Hackney Wick co-design workshop, for example, several older adults suggested introducing a system of “walkways in the sky” within the neighborhood in which we worked that connected points of interest. Mitchell and Nørgaard [30] reported how the playful atmosphere produced by a sketching activity led to novel design ideas from older adults engaged in a participatory design exercise. Frohlich and colleagues [12] found that older adults demonstrated “considerable creativity” when asked to re-design information and communication technology (ICT) product concepts, proffered by the researchers, in a focus group setting. Davidson and Jensen found that some older adults produced creative designs when involved in co-design activities, including low-fidelity prototyping employing various materials [29]. However, the designs produced by others were judged to demonstrate little creativity [29], while some researchers suggested that older adults make better critics of existing designs than originators of new designs [22].

Our findings on the usefulness of Talking Mats^®^ as a tool for eliciting views accords with the experiences reported by others. However, whereas most researchers typically employed the technique with, as was intended by its creators [26], population groups presenting communication difficulties and/or cognitive impairments, such as older adults with dementia [26,27], people with learning disabilities [54,55], people with intellectual disability [56], and people with Huntington’s Disease [57], we employed it both with older adults who presented these conditions and with those who did not. It proved a highly effective way to explore, structure, and support the expression of views with all participants, particularly amongst those who did not present communication difficulties and/or cognitive impairments. Given its use of colorful cartoon illustrations, intentional simplicity, and game-like qualities, there was some concern that there might have been reluctance on the part of some of these older adults to participate. In their research, Xie and colleagues [53] found that some older adults disliked participatory design techniques that they perceived as being exercises in “playing not working”. Our concerns were, however, entirely unfounded. The older participants enthusiastically engaged in the activity and gave careful thought to where they positioned the different aspects of the home and outdoor environment on the visual scale. Insights into how they encountered, used, and evaluated multiple aspects of the home and of outdoor environments were revealed, providing valuable information for environmental design decisions. This technique seems to offer rich opportunities for further use among more diverse population groups.

*Limitations* of our work include the relatively small numbers of participants engaged in the co-design events and one-to-one interactions, and the relatively small (although diverse) number of contexts within which the engagement techniques were trialed. Despite extensive recruitment efforts, including advertising in local media, engaging with charities, community groups, networks associated with older adults, and working with health practitioners, recruiting participants proved challenging. Time and resource pressures placed limits on the number and scale of events we could deliver. Future iterations of the work could usefully implement a set of novel engagement techniques in a wider range of contexts with a larger number of diverse older adults. This could support firmer conclusions on the potential for more novel forms of engagement to facilitate the involvement of diverse older adults in environmental design decisions.

## 5. Conclusions

The experience of employing a range of eight, relatively novel engagement techniques with older adults in a series of co-design workshops, design review events, and one-to-one interactions indicates that there are multiple potential ways to involve this age group in designing age-friendly homes and neighborhoods. Based on our experiences, we offer the following practical recommendations to others interested in designing age-friendly homes and neighborhoods with older people:

*1. Recommended tools and techniques:* Given their value as independent and combined instruments, for place-based projects, we recommend using walking interviews in conjunction with photovoice to see and experience environments from the perspective of older adults. These techniques could, for instance, be used to ensure neighborhood revitalization projects take account of older adults’ requirements and preferences, or that the development of built environment policy and standards, such as future iterations of England’s Decent Homes Standard [58] and Building Regulations [59], adequately respond to older adults’ needs. To help elicit older adults’ environmental preferences, attitudes to the built environment, and so on, we recommend introducing images into conversations. Both photo-elicitation, using researcher-generated images, and Talking Mats^®^ proved highly effective at stimulating, structuring, and, in the case of the latter, providing a visual record of older adults’ views. To quickly and clearly communicate environmental design ideas to older adults, we recommend using models, while, to generate design ideas from older adults, model-making was shown to be effective in our work.

*2. Actions to encourage older adults’ enthusiastic and meaningful participation:* The following factors might help to secure older adults’ involvement in design activities and maximize the value of their contributions: a welcoming, convivial, supportive atmosphere; knowledgeable and empathetic facilitators; refreshments and ample breaks; a convenient time and venue—the latter accommodating visual, aural, and mobility limitations; varied ways to engage—group settings, one-to-one interactions, drawing, talking, etc.; opportunities for individuals to control the length and level of their participation and genuine recognition of the value and (often limited) availability of participants’ time when planning participatory design activities.

*3. Leave preconceptions at the door:* Our preconceptions about how different techniques might function and might be received by older adults, as well as about the type of design ideas that older adults might contribute, frequently proved false. Talking Mats^®^ proved to be a highly effective and positively received way to explore, structure, and support the expression of views amongst community-dwelling older adults presenting no communication difficulties or cognitive impairments. Older adults contributed often bold and creative design suggestions that moved far beyond “traditional” age-friendly design “solutions”. Although the process and outputs often proved surprising, the unique benefits that participatory design can bring to both the object of and the participants in design decisions lead us to recommend consideration of the approach to designers, planners, developers, and others interested in creating age-friendly homes and neighborhoods.

## Figures and Tables

**Table 1:** Demographic data for the four study areas.

Location		Central Manchester	Edinburgh	Hackney Wick	Kirkwall
Relevant Local Authority		Manchester	Edinburgh	Hackney	Orkney Islands
Total population (2018)	Pers.	547,600	518,500	279,700	22,200
Age 60 and over	Pers.	13%	20%	10%	27%
Mean age		33	39	32	43
Very bad health	Pers.	2%	1%	2%	1%
Bad health	Pers.	5%	3%	5%	2%
Fair health	Pers.	12%	10%	11%	10%
Good health	Pers.	32%	29%	31%	32%
Very good health	Pers.	49%	58%	52%	54%
Unemployed (2018/2019)	Pers.	5%	4%	5%	2%
Retired (2018/2019)	Pers.	3%	15%	No estimate	No estimate
No qualifications (2018)	Pers.	11%	6%	12%	No estimate
Detached house	Hhs.	5%	11%	2%	61%
Semi-detached house	Hhs.	31%	13%	4%	24%
Terraced house	Hhs.	30%	13%	16%	8%
Flat	Hhs.	33%	64%	78%	7%
Owner occupied	Hhs.	38%	60%	24%	71%
Social rented	Hhs.	32%	17%	44%	14%
Private rented	Hhs.	28%	22%	29%	11%

Data relate to 2011 unless otherwise stated. Health data are self-reported. Data provided at local authority level. Pers. refers to persons. Hhs. refers to households. Source: Office for National Statistics.

**Table 2:** Participatory design events.

Location	Event Type	Event Length	Size (*n*)	Recruited Characteristics	Recruitment Pathways	Engagement Techniques ^1^	Evaluation Methods
Manchester (Year 1)	Co-design workshop	3 days	12	Community dwelling older adults	Older adult groups and networks	Photovoice, walking interviews, photo-elicitation	Observation, participant self-complete survey, informal discussion with participants
	Design review	1 day	6	Community dwelling older adults	Invitation to co-design workshop participants	Town hall meeting	Observation, participant self-complete survey, informal discussion with participants
Edinburgh (Year 1)	1-to-1 interactions	1–1.5 h	22	Community dwelling older adults, stroke survivors, people with dementia	Older adult groups and networks, community groups, local advertising, health practitioners	Photo-elicitation, Talking Mats^®^	Observation, informal discussion with participants
Hackney Wick (Year 2)	Co-design workshop	2 days	13	Community dwelling older adults	Older adult groups and networks, community groups, local advertising	Photovoice, model-making, walking interviews, participatory mapping, drawing	Observation, participant self-complete survey, informal discussion with participants
	Design review	1 day	18	Community dwelling older adults	Invitation to co-design workshop participants, older adult groups and networks, community groups, local advertising	Design fair	Observation, informal discussion with participants, comment cards
Kirkwall (Year 3)	Co-design workshop	1 day	11	Community dwelling older adults	Older adult groups and networks, community groups, local advertising	Model-making, participatory mapping	Observation, informal discussion with participants, comment cards
	Design review	0.5 days	11	Community dwelling older adults	Invitation to co-design workshop participants, older adult groups and networks, community groups, local advertising	Design fair	Observation, informal discussion with participants, comment cards

^1^ Focusing on the techniques discussed within the article.

**Table 3:** Central Manchester co-design workshop: participant perspectives on engagement techniques.

Item	Agreement with Statement: 1 Strongly Disagree to 5 Strongly Agree					
	*n*	1	2	3	4	5
***Walk and talk: encompassing walking interviews and photovoice***						
I enjoyed taking part	11			9%	18%	73%
I was able to discuss topics which I feel are important to the design of environments	10				20%	80%
It was an effective method for exploring my views on the design of environments	10			10%	20%	70%
***Photoelicitation***						
I enjoyed taking part	10				50%	50%
I was able to discuss topics which I feel are important to the design of environments	9			11%	22%	67%
It was an effective method for exploring my views on the design of environments	9			11%	11%	78%

Notes: Not all participants completed the survey/completed the survey in full.

**Table 4:** Hackney Wick co-design workshop: participant perspectives on engagement techniques.

Item	Agreement with Statement: 1 Strongly Disagree to 5 Strongly Agree					
	*n*	1	2	3	4	5
***Walk and talk: encompassing walking interviews and photovoice***						
I enjoyed taking part	7				29%	71%
The activity was easy to understand	7				29%	71%
It was easy to take part in the activity	7				29%	71%
The activity provided opportunities for me to highlight topics which I feel are important to the design of environments	6				33%	67%
The activity was an effective method for exploring my views on the design of environments	7			14%	29%	57%
***Mood cards and mood map: encompassing participatory mapping and drawing***						
I enjoyed taking part	6			17%	33%	50%
The activity was easy to understand	6				50%	50%
It was easy to take part in the activity	6				50%	50%
The activity provided opportunities for me to highlight topics which I feel are important to the design of environments	6				50%	50%
The activity was an effective method for exploring my views on the design of environments	6				33%	67%
***Save it/change it: encompassing drawing***						
I enjoyed taking part	9	11%		11%		78%
The activity was easy to understand	8			13%	13%	75%
It was easy to take part in the activity	8			13%		88%
The activity provided opportunities for me to highlight topics which I feel are important to the design of environments	9			22%	33%	44%
The activity was an effective method for exploring my views on the design of environments	8			38%	25%	38%
***Master planning: encompassing model-making and drawing***						
I enjoyed taking part	9			11%	22%	67%
The activity was easy to understand	9			11%	22%	67%
It was easy to take part in the activity	9			11%	11%	78%
The activity provided opportunities for me to highlight topics which I feel are important to the design of environments	8			25%	25%	50%
The activity was an effective method for exploring my views on the design of environments	8			25%	25%	50%

Notes: Not all participants completed the survey/completed the survey in full.
